# The Effects of Passive Leg Raising and Maintenance Fluid Administration on Pulse Oximetry Waveform Amplitude and Peak Variability in Mechanically Ventilated Patients in Sepsis and Septic Shock

**DOI:** 10.3390/diagnostics15070798

**Published:** 2025-03-21

**Authors:** Jamie Kagihara, Xinning Guo, Ahmet Baydur

**Affiliations:** 1 Los Angeles General Medical Center, Los Angeles, CA 90033, USA; jamie.m.kagihara@gmail.com; 2 Keck School of Medicine, University of Southern California, Los Angeles, CA 90033, USA; 3 Viterbi School of Engineering, University of Southern California, Los Angeles, CA 90089, USA; xinningg@usc.edu

**Keywords:** intrathoracic pressure, intravascular volume, mechanical ventilation, passive leg raising, pulse oximeter waveform variation, tidal volume, volume resuscitation

## Abstract

**Objective:** We sought to assess variations in pulse oximetry waveform amplitude (ΔP) and peak values (ΔS) separately during passive leg raising (PLR) and challenge plus maintenance crystalloid volume resuscitation over time in mechanically ventilated (MV) patients in shock. **Methods***:* Variables were recorded and analayzed using previously described techniques. Findings were compared between the following: at baseline, during passive leg raising (PLR), with 0.9% normal saline administration (or removal), and applying tidal volume (Vt), peak, and mean airway pressure (Paw,peak and Paw,mean, respectively) and positive end-expiratory pressure (PEEP) as covariates in multifactorial logistic regression analysis. **Results***:* Twenty patients with sepsis or septic shock were included in the analysis. Origins of sepsis varied. Their diagnoses upon admission to the intensive care unit included sepsis in nine (45%), septic shock (defined as the need for vasopressors) in nine (45%), and one (5%) rescuscitated from pulseless electrical activity following heroin overdose, all of whom were supported by volume control MV. Eleven patients required vasoactive drugs at the outset, of which seven were on norepinephrine. Three patients required surgical drainage or removal of necrotic tissue. Median ΔP and ΔS decreased, respectively, by 42% and 37% with PLR (*p* = 0.036 and *p* = 0.061, respectively). There were no significant changes in ΔP and ΔS between PLR and net fluid volume administered. Correction for body weight did not change these relationships. Application of Vt, Paw,peak, Paw,mean, and PEEP did not significantly influence these changes. **Conclusions:** Hemodynamic repsonse to slow fluid volume administration can be assessed by changes in the pulse oximetry waveform amplitude over time. The effects of mechanical ventilation are negligible.

## 1. Introduction

Pulse oximetry (photoplethysmography) has the advantage over traditionally used methods of estimating intravascular volume (volume responsiveness) (such as pulmonary and systemic arterial monitors) of being noninvasive, convenient, and inexpensive [1,2]. Its waveform fluctuations have been shown to correlate with intraarterial waveforms [3,4,5,6,7,8,9,10]. Pulse O_2_ saturation (SpO_2_) waveforms correlate with intravascular volume status and volume responsiveness [1,2,3,4,5,6,7,8,9]. Delerme et al. [7], in a study of pulse oximetry waveform amplitude variation (ΔPOP) in 25 spontaneously breathing volunteers recorded at several time points, found that PLR induced a significant decrease in ΔPOP. In a later study, however, the same group [8] found a weak correlation between cardiac index and ΔPOP variations with PLR (*r* = 0.40; *p* < 0.01). Mechanical ventilation introduces major changes in cardiac function and blood pressure caused by impedance of blood return to the right heart as intrathoracic pressure rises [11,12]. Most studies have assessed arterial pulse wave and oximetric wave variations during fluid rapid volume infusions (challenges or boluses) during early sepsis and septic shock [1,2,3,4,5,6,7,8,9]. We found no studies assessing changes in plethysmographic pressure variations by oximetry during maintenance volume administration (with or without volume challenge) over time. Rapid infusions over short periods, while potentially life-saving and posessing the ability to stabilize hemodynamic properties, may be disadvantageous under certain situations, such as heart failure.

The primary aim of this study was to assess changes in pulse oximetry waveform amplitude at baseline and separately during passive leg raising (PLR) and volume replacement over time (including non-challenge) in mechanically ventilated (MV) patients in respiratory failure and sepsis or septic shock. A second aim was to determine the effects of positive pressure ventilation on these changes. We hypothesized that the amplitude and peak-to-peak variability of the SpO_2_ waveform (ΔP and ΔS, respectively) decrease during PLR and volume replacement (combining challenge and maintenance fluid administration) in mechanically ventilated patients as they do in spontaneously breathing individuals. Measurements included changes in ΔP and ΔS separately following PLR and total fluid administration over a period of time.

## 2. Methods

### 2.1. Patients

Pulse oximetry waveform analysis was conducted in patients aged 18–80 years admitted to the medical intensive care unit (MICU) and being treated for management of hypoxemic respiratory failure. Inclusion criteria required that patients have sepsis and/or septic shock diagnosed on admission. Sepsis was defined according to Singer et al. [13]. The study was conducted in accordance with the Declaration of Helsinki and approved by the Institutional Review Board of the University of Southern California Medical Center (HS-16-00873). Data obtained included anthropometric characteristics, diagnoses, vital signs, ventilator modes and settings, net volume of fluid administered (all normal saline) or removed (including during hemodialysis), and inotropic and vasopressor agents administered. Intravenous fluid was administered as long as the patient exhibited improvement in hemodynamic variables, as described in the 2016 Surviving Sepsis Guidelines [14]. Patients were given fluids to replace recorded losses or correct dehydration or electrolyte abnormalities. Maintenance fluids were continued as per the intensive care unit protocol of this institution. Patients were excluded if they were undergoing cardiopulmonary resuscitation, were hypothermic (core temperature < 32 °C [89.6 °F]), had intra-abdominal hypertension, were receiving extracorporeal membrane oxygenation or mechanical circulatory support, or had evidence of mitral or severe tricuspid valve dysfunction confirmed by echocardiography.

### 2.2. Procedure and Measurements

Pulse oximetry tracings were obtained by multichannel physiologic recording (telemetry) and printed out on paper strips for subsequent analysis (IntelliVue X2, Philips, North America Corporation, Andover, MA, USA). Recordings were made with subjects in semi-recumbent position. The patients were sedated with fentanyl, midazolam, and/or propofol while receiving ventilatory support [Richmond Agitation–Sedation Scale (RASS) −3 to −4] [15]. No recruitment maneuvers were conducted during pulse oximetry variation measurements. Recordings were made only if the quality of the signal was optimal according to the perfusion index displayed on the monitor.

Oximetry tracings were obtained over 5 respiratory cycles at baseline. This was followed by a PLR test performed by raising the patient’s legs to a 45° angle while the patient’s trunk was lowered from a semi-recumbent to supine position with no changes in the hip angle, using the bed’s position-changing mechanism [7]. A third set of oximetry tracings (without performing PLR) was obtained between 20 and 81 h later. The total volume of crystalloid administered or removed was determined by the primary care providing team, recorded in L, and net volume administered or removed per unit time was recorded in mL/h.

### 2.3. Data Analysis

**Figure 1:**
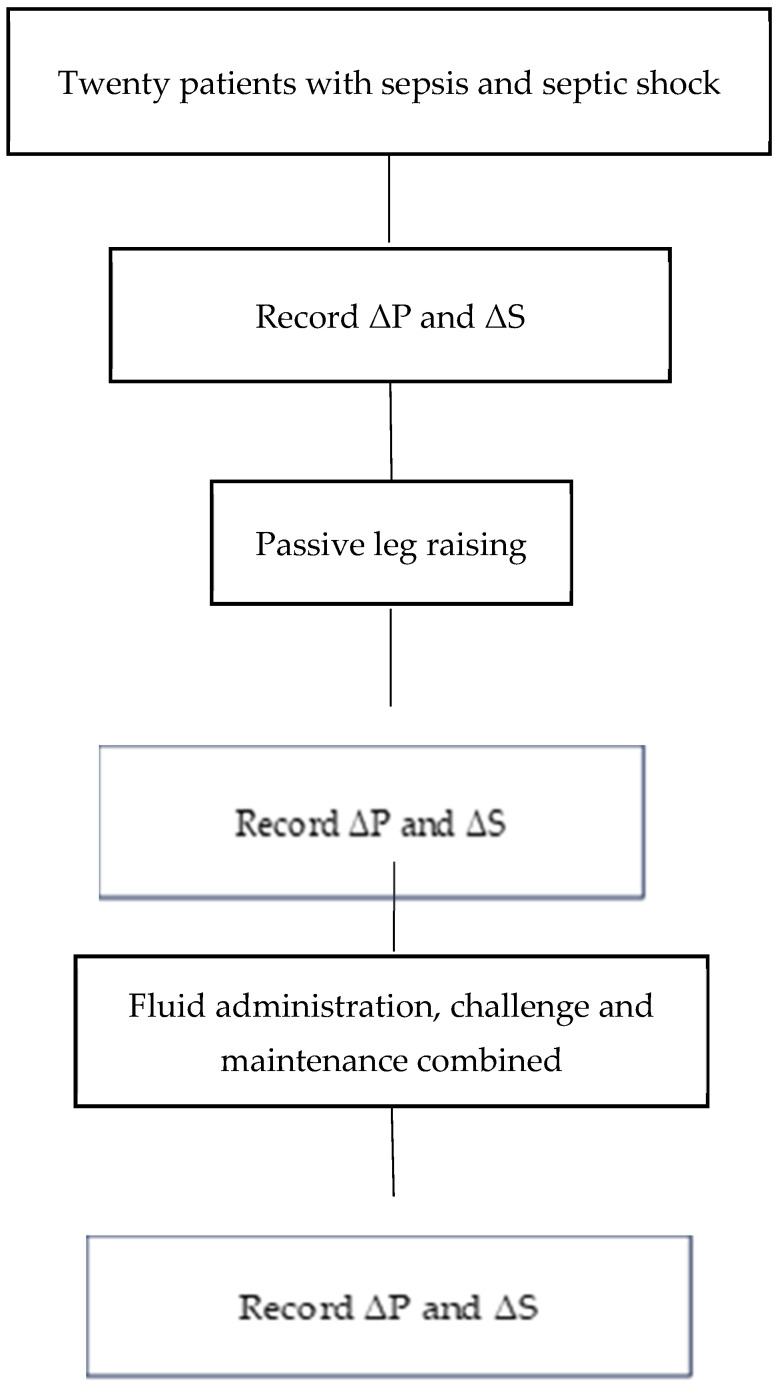
Flow chart of procedure.

Variation in the amplitude (ΔP) of the SpO_2_ wave was determined as the difference between end-inspiration and end-expiration over five respiratory cycles in SB and MV patients (Figure 1) [16]:

ΔP = (amp_max_ − amp_min_)/{(amp_max_ + amp_min_)/2} × 100 (1)

The variation in the peak values (ΔS) of the SpO_2_ waveform at end-inspiration and end-expiration in SB and MV patients was expressed as (Figure 1):

ΔS = (peak_max_ − peak_min_)/{(amp_max_ + amp_min_)/2} × 100 (2)

where amp = amplitude.

Values for ΔP and ΔS were calculated before and after passive leg raising and separately following net intravascular fluid volume administered or removed. The relation of net fluid volume change to changes in ΔP and ΔS was assessed following each intervention.

### 2.4. Statistical Analysis

Values were expressed as mean (SD) or as median (interquartile range), depending on the normality of distribution of the cohort data. Associations between changes in ΔP and ΔS and net fluid administered (or removed) were assessed by Pearson correlation [17]. In addition, multivariate regression analysis by separately incorporating tidal volume (Vt), peak airway pressure (Paw,peak), mean airway pressure (Paw,mean), and positive end-expiratory pressure (PEEP) was conducted. We utilized the Kruskal–Wallis H test to compare the 3 groups in a non-parametric context. To determine which specific groups showed the greatest differences, the Mann–Whitney U test was used for pairwise comparisons.

## 3. Results

Demographic and clinical data for 20 randomly selected patients in sepsis and receiving mechanical ventilatory support are summarized in Table 1. Patients were included in the study within 24 h of the diagnosis of sepsis. Fifty-three percent of the patients were Latino, 25% Caucasian, 15% Asian, and 7% African-American. Their diagnoses upon admission to the intensive care unit included sepsis in nine (45%), septic shock (defined as the need for vasopressors) in nine (45%), and one (5%) rescuscitated from pulseless electrical activity following heroin overdose, all of whom were supported by volume control MV. Additional details, including secondary diagnoses, are provided in Table 1. Eleven patients were receiving vasoactive drugs, of which seven were receiving norepinephrine. By the third set of measurements (post-fluid administration, total volume), only five remained on norepinephrine. Mean (±SD) total fluid volume administered was 6.2 ± 4.3 L (152 ± 117 mL/h) over 30.4 ± 16.1 h, of which 43.7% was administered within the first 24 h (including challenge volumes).

The following three patient cases required surgical drainage or removal of necrotic tissue: the retropharyngeal abscess, the infected hip arthroplasty, and the Fournier’s gangrene.

**Figure 2:**
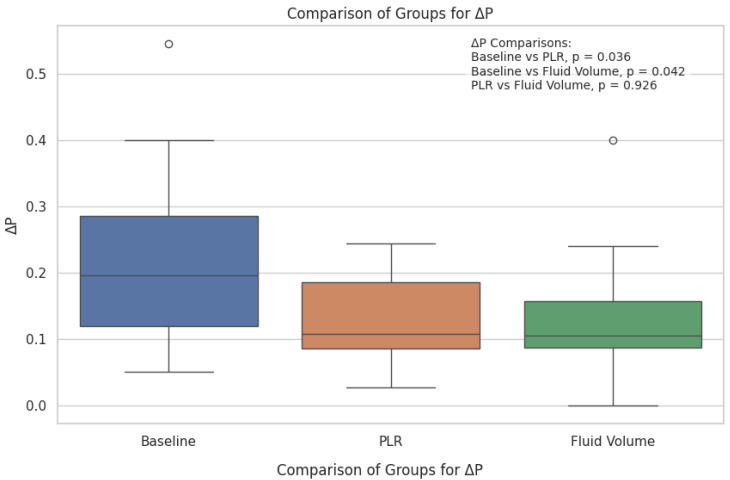
Respiratory variations of pulse oximetry wave amplitude (ΔP) following passive leg raising (PLR) and net post-fluid volume administration. Values represent median [IQR]. PEEP, positive end-expiratory pressure; PLR, passive leg raising; Vt, tidal volume. Kruskal–Wallis H test used for comparison of more than two groups for non-parametric distribution. Box plots represent median {IQR]. Open circles represent outliers.

**Figure 3:**
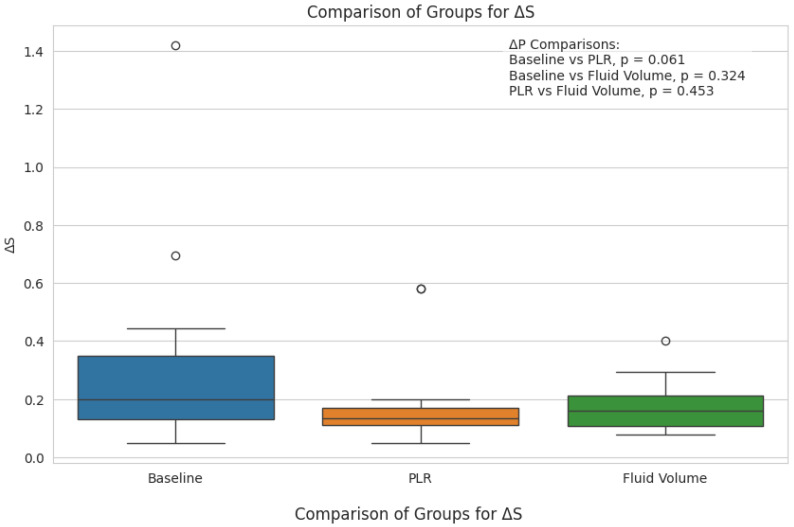
Variations of pulse oximetry wave peak (ΔS) following passive leg raising (PLR) and net post-fluid volume administration. Box plots represent median {IQR]. Open circles represent outliers.

**Figure 4:**
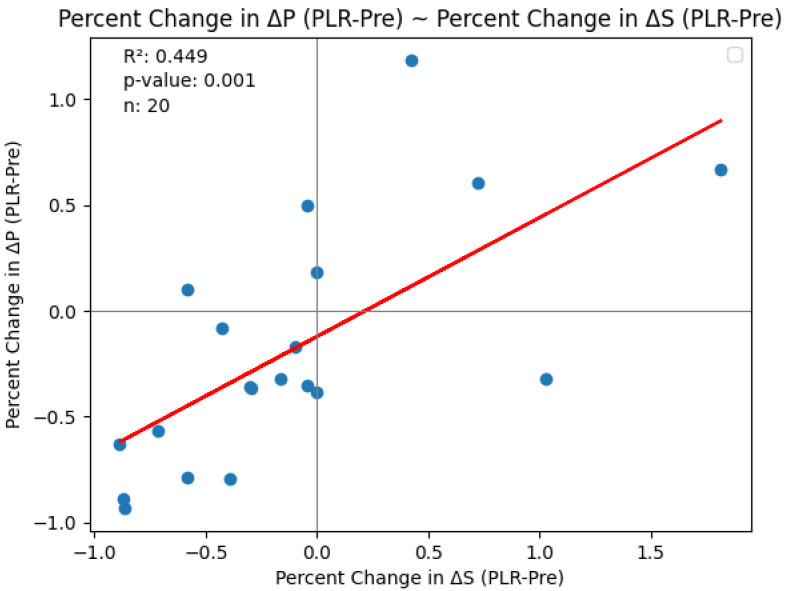
Association of % change in pre-PLR ΔP with % change in pre-PLR ΔS.

**Figure 5:**
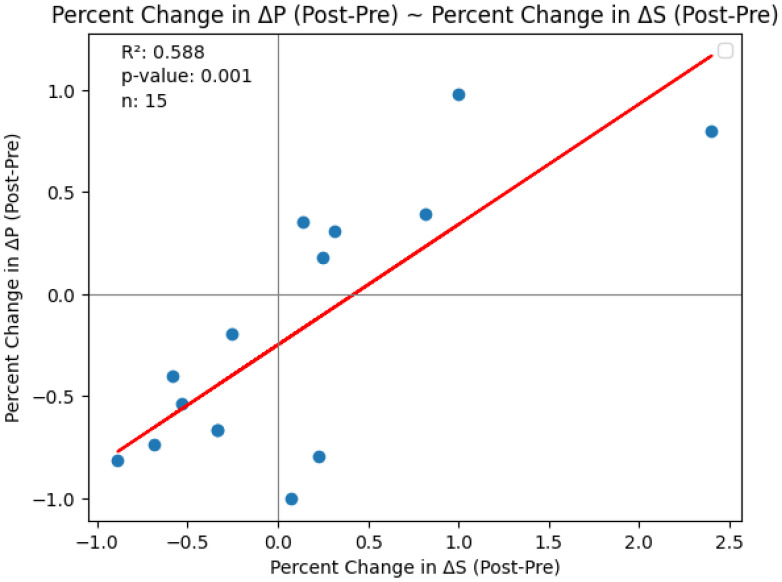
Association of % change in post-PLR ΔP with % change in post-PLR ΔS.

**Figure 6:**
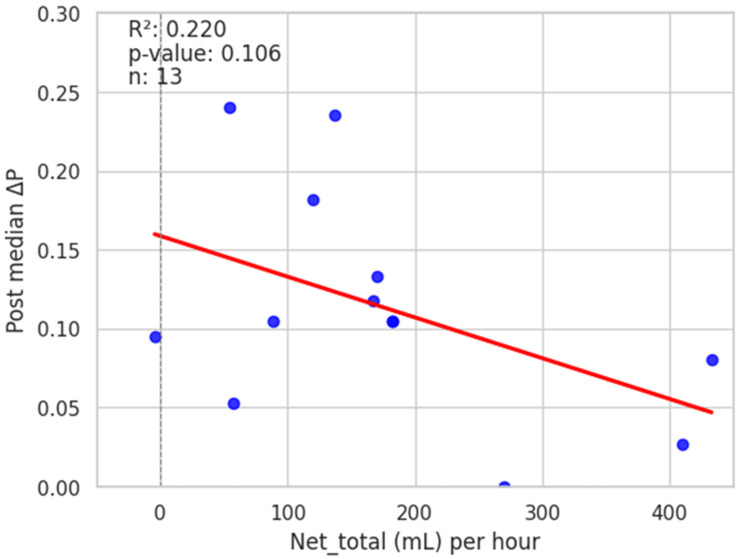
Relation between net total fluid administration per hour and change in pulse wave amplitude, ΔP.

Pulse oximetry was recorded at the fingertip and earlobe in 19 patients and 1 patient, respectively. Pre- and post-PLR ΔP and ΔS data for changes were available for all 20 patients; net post-fluid administration data were available for 15 patients. Thirteen patients (65%) exhibited a mean (± SD) decrease of 51 ± 26% in ΔP from baseline with PLR; in eight (62%) of these patients, ΔP remained unchanged at the end of volume administration. Delta S decreased by 42 ± 29% following PLR in 13 (65%) patients; in 7 (54%) of these patients, ΔS remained unchanged following volume administration. Figure 2 and Figure 3 show the overall effect of PLR on median ΔP and ΔS, respectively. Median ΔP and ΔS decreased by 42% and 37%, respectively, with PLR (*p* = 0.036 and *p* = 0.061, respectively). There were close associations between % change in pre-PLR ΔP with % change in pre-PLR ΔS (Figure 4) and between % change in post-PLR ΔP with % change in post-PLR ΔS (Figure 5). Associations between ΔP and ΔS and net fluid volume administered over time following PLR were poor. The closest associative trend was between ΔP and net fluid volume administered (*p* = 0.106, Figure 6). Correction for body weight, Vt, respiratory rate, Paw,peak, Paw,mean, and PEEP as covariates did not exert significant influence on these relationships. In addition, the administration of vasoactive drugs did not influence which patients responded to PLR or net volume administration over time. Finally, there were no associations between changes in heart rate or blood pressure with changes in ΔP and ΔS during PLR and volume administration.

## 4. Discussion

As far as we know, this is the first study assessing the effects of slow (maintenance with or without challenge) volume administration over time on pulse waveform amplitude and peak variations. In this study, we assessed the effects on the pulse oximetry waveform configuration of PLR separately, followed by 0.9% saline administration (challenge plus maintenance combined) over time in patients with sepsis and septic shock receiving mechanical ventilatory support. The main findings were as follows: (a) a significant decrease in ΔP and a trend for decrease in ΔS following PLR but not with net crystalloid volume administered following PLR, and (b) differences in ventilator settings did not significantly affect changes in ΔS and ΔP.

The absence of significant differences in ΔP and ΔS between PLR and volume administration per unit time (challenge plus slow infusion) indicates that volume response had diminished by the time patients received continued fluid infusion following PLR. This finding should not be considered as exhibiting the same effect as administering a rapid bolus of fluid (e.g., 500 mL over 15–30 min) early in the management of sepsis [18]. PLR results in a 250 to 350 mL transfer of blood volume from the legs to the thoracic circulation [4]. In this connection, observational studies and randomized trials have shown that liberal fluid strategies have been associated with harm, with increased risk of end-organ injury and death [19,20,21,22], although studies in general have been considered to be of low quality evidence [23]. The CLASSIC trial [24] showed no difference in 90-day mortality or adverse events amongst patients receiving restricted or liberal fluid therapy. The total volume administered over 24 h in the restricted fluid group was <50% of the volume administered in the liberal fluid group. While we did not assess clinical outcome, we found a significant negative relationship between ΔP and net volume administered and a similar trend for ΔS over periods of up to 81 h, indicating that pulse pressure variation can be detected beyond the initial fluid challenge in septic patients.

Respiratory variations of pulse oximetry plethysmographic waveform amplitude closely associate with respiratory variations in arterial pulse pressure and are sensitive to changes in ventricular preload [1,2,3,4,5,6,7,8,9,10]. A PLR-induced decrease in pulse pressure variation (PPV) reflects a reduction in preload responsiveness induced by the preload challenge and detects preload responsiveness [11]. The 65% prevalence in response (decrease) to PLR in ΔP and ΔS exhibited by our patients is similar to the 58% response reported by Delerme et al. [7] and 61% response reported by Mallat et al. [5]. The overall mean decrease of 42% in ΔP following PLR is also similar in magnitude to those found by others measuring variations in pulse oximetry waveform amplitude (ΔPOP) following fluid challenge, a 31% decrease reported by Delerme et al. [7] and 42% reported by Canneson et al. [6]. In this connection, a recent meta-analysis of 17 studies by Chaves et al. [25] described pulse pressure variation, stroke volume variation, pulse variability index, and PLR to be superior to central venous pressure and the inferior vena cava collapsibility index.

The relation between variations in intraarterial blood pressure and pulse oximetery wave amplitude and peak is often not in phase. In a previous study [16], we found in 12 patients the phase angle between the intraarterial and pulse oximetry waveforms to be (mean ± SD) 79 ± 22 degrees, indicating that placement of the sensor on the fingertip resulted in the waveforms being out of phase from the radial arterial pulse tracing by 0.15 sec. Variability of ΔP and ΔS are directly related to the number of respiratory cycles analyzed [16]. In this study, ΔP and ΔS were averaged from five respiratory cycles. We found large inter-subject coefficients of variation (c.v.) in ΔP and ΔS; c.v. for ΔP had a range of 54–111%, while c.v. for ΔS had a range of 63–119%. The intra-individual c.v. for ΔP had a range of 12–115%; for ΔS, it had a range of 20–102%. These findings reflect the many factors affecting variability of measurements even within subjects: age, varying volume of crystalloid infusion, mechanical ventilation, hemodynamics, and anatomical location of the oximeter sensor [26,27]. In a study of 28 anesthetized subjects, Desgranges et al. [27] showed that forehead placement of the sensor provided the highest sensitivity and specificity (89% and 78%, respectively) to predict fluid responsiveness, as confirmd by hemodynamic methods, compared to placement on the ear (74% and 74%, respectively) or finger (74% and 67%, respectively).

Patients were evaluated while receiving mechanical ventilation in volume control mode. Variability in pulse oximetry waveforms has yet to be evaluated in other ventilation modes. Previous studies have shown that driving pressure (difference between airway plateau pressure and PEEP) is more closely related to pulse waveform amplitude and baseline fluctuations than mean or plateau pressure alone [28]. Documentation of plateau and driving pressures were available in only a few of our patients, so we did not consider driving pressure in our analysis.

### Limitations: Use of Other Measures of the Effects of Systemic Perfusion

This study had some limitations. First, this was a single-center study with a small number of patients, but it was intended as a pilot project. Second, we did not measure hemodynamic indices such as the left ventricular ejection fraction (LVEF), peripheral pulse index (PPI), cardiac output, or systemic vascular resistance, measurements that would have confirmed that changes in ΔP and ΔS were associated with hemodynamic changes. There are, however, limitations to the PPI. Lima et al. [29] found that PPI distribution in a normal population is highly skewed. Delerme et al. [8] found a weak association between cardiac index and ΔPOP variations with PLR (*r* = 0.40; *p* < 0.01). Second, PPI analysis is sensitive to body movement and is reduced by factors including stress and anxiety that result in sympathetic activation and peripheral vasoconstriction [30,31]. Nevertheless, all of our patients were heavily sedated (RASS −3 to −4) and paralyzed, so patient movement was not an issue. Pulse oximeter wave analysis has also been shown to be more accurate in patients with higher perfusion status [32].

Pulse pressure variation has also been shown to be partially unreliable in predicting volume responsiveness in patients with ARDS ventilated according to the recommended lung protective strategy, which entails delivery of Vt of 6 mL/kg or less [26,27]. Although a PPV > 10–12% is reliable, a lower PPV (<10%) may fail to detect response to volume administration, although use of the driving pressure may circumvent this challenge. Thus, performance of alternative preload responsiveness tests such as passive leg raising or end-expiratory occlusion tests may be necessary in the presence of low PPV values. Finally, we manually recoded pulse pressure variation values displayed on the Philips^®^ IntelliVue X2 without using dedicated software.

Finally, we did not compare the effect of placing the oximeter sensor at different locations in this study. We had evaluated this issue in a previous publication [16]. Nineteen of twenty patients had the sensor placed on their finger and only one on the forehead, so a comparison of recording sites was not possible here. Previous authors, however, have discussed the effects of different sensor locations on waveform analysis [26,27].

## 5. Conclusions

The hemodynamic repsonse to slow fluid volume administration can be assessed by changes in the pulse oximetry waveform amplitude over time. The effects of mechanical ventilation are negligible. Future studies are needed to confirm the usefulness of these findings during slow fluid administration (with or without challenge) and should include simultaneously recorded direct (invasive) measurements of cardiac output and index, stroke volume, PPV, and PPI, while taking into account their own intrinsic limitations.

## Figures and Tables

**Table 1:** Baseline anthropometric and clinical data in 20 patients.

Age	58.7 ± 18.3
Sex (M/F)	13/7
BMI (kg/m^2^)	26.5 ± 10
Temperature (°C)	37.1 ± 14.6
Heart rate (beats/min)	94.3 ± 27.6
Blood pressure (syst/diast, mm Hg)	104 ± 23/68 ± 12
MAP (mm Hg)	80.1 ± 14.6
Respiratory rate (on ventilator, breaths/min)	19.1 ± 4.1
SpO_2_ (%)	98.4 ± 1.6
FiO_2_	0.59 ± 0.23
Tidal volume (mL)	455 ± 37
Respiratory rate (breaths/min)	19.3 ± 3
Peak inspiratory flow (L/min)	49.2 ± 8.4
Peak airway pressure (cm H_2_O)	25.2 ± 7.5
Mean airway pressure (cm H_2_O)	10.6 ± 2.5
Positive end-expiratory pressure (cm H_2_O)	5.9 ± 1.6
Airway resistance (cm H_2_O/L/s) ***	21.3 ± 7.1
Total net volume crystalloid (mL)	3954 ± 2734
Net intravascular crystalloid administered (mL/h)	152 ± 12
Number of patients receiving vasoactive drugs at baseline:	
norepinephrine	7
epinephrine	2
vasopressin	2
**Individual diagnoses:**	
Primary (source of sepsis)	Secondary
Sepsis (aspiration PNA)	Upper GI bleed
Septic shock (multifocal PNA)	AKI
Septic shock (GI bleed)	Alcoholic cirrhosis
Septic shock (retropharyngeal abscess)	Uncontrolled DM
Sepsis (respiratory failure, PNA)	Encephalopathy
Septic shock (PNA)	HRF
Sepsis (aspiration PNA)	AKI
Septic shock (aspiration PNA)	HRF
Septic shock (neutropenic fever)	Testicular CA
Septic shock (infected hip arthroplasty)	Uncontrolled DM
Septic shock (cholecystitis)	Encephalopathy
S/P PEA Arrest	Heroin Overdose
Sepsis (skin and soft tissue infection)	HRF
Sepsis (aspiration PNA)	Multiple myeloma,
s/p auto-SCT	
Septic shock (PNA)	Adenoca, lung
Sepsis (aspiration PNA)	Vascular dementia
Sepsis (aspiration PNA)	AIDS
Sepsis (pneumonia)	Hyponatremia
Sepsis (Fournier’s gangrene)	AKI
Sepsis (pyelonephritis)	Alcohol withdrawal

Abbreviations: AIDS, acquired immune deficiency syndrome; AKI, acute kidney injury; BMI, body mass index; CA, cancer; DM, diabetes mellitus; FiO2, fraction of inspired oxygen concentration; GI, gastrointestinal; HRF, hypoxemic respiratory failure; MAP, mean arterial pressure; PEA, pulseless electrical activity; PNA, pneumonia; auto-SCT, autologous stem cell transplant; S/P, status-post; SpO_2_, pulse oximetry O_2_ saturation. Values represent mean ± SD. *** Includes resistance of endotracheal tube.

## Data Availability

The original contributions presented in this study are included in the article. Further inquiries can be directed to the corresponding author.
